# Experimental Investigation of Inductive Sensor Characteristic for Blade Tip Clearance Measurement at High Temperature

**DOI:** 10.3390/s19173694

**Published:** 2019-08-25

**Authors:** Zhenxia Liu, Ziyu Zhao, Yaguo Lyu, Lingqiang Zhao

**Affiliations:** School of Power and Energy, Northwestern Polytechnical University, Youyi West Road 127#, Xi’an 710054, China

**Keywords:** inductive sensor, tip clearance, repeatability error, high temperature, calibration

## Abstract

Turbine tip clearance of aero-engine is important to engine performance. Proper tip clearance can reduce the gas leakage over turbine blade tips and improve the engine efficiency of turbo machinery and reduce the fuel consumption. Therefore, accurate tip clearance measurement is essential. The inductive measurement method is one of the non-contact distance measurement methods, which has the characteristics of high sensitivity, fast response speed, and strong anti-interference ability. Based on the principle of inductive sensor measuring tip clearance, the ambient temperature change may cause the material electromagnetic performance change for the conductivity and permeability varies with temperature. In order to verify the temperature effect on the sensor performance, the repeated calibration experiments were carried out to obtain the sensor repeatability error of 5.4%. Then, the sensor was calibrated in the range of 0 mm–4 mm clearance at temperature from 600 °C to 1000 °C and obtained the measurement error of 4.6%. Results indicate when the temperature ranged from 600 °C to 1000 °C, clearance measurement error is smaller than the sensor repeatability error so the temperature effect on the sensor characteristics can be ignored. This conclusion makes the sensor promising for monitoring the blade tip clearances at various temperature environment.

## 1. Introduction

The blade tip clearance of gas turbine is significant for its performance and efficiency. Therefore, precise measurement of tip clearance is the premise of accurate design and optimization of the tip clearance [1,2,3]. The study of sensor with high precision and high resolution for tip clearance measurement is necessary and crucial. Numerous non-contact measurement technologies are developed, including microwave method, optical method, capacitive method, and inductive method.

Mark R.W. et al. [4,5,6] from NASA Glenn Research Center started effort on microwave method applying to tip clearance measurement since 2003. The microwave sensor probe is able to operate at extremely high temperature and is unaffected by the contaminant in the turbine. While the sensing range is limited by the working frequency and the probe can only operate at 900 °C without cooling. As early as 1982, NASA and GE published their cooperative research results of an optical sensor for measuring tip clearance, including test results on the compressor disk [7]. Since 2013, García I. and Zubia J. et al. from University of the Basque Country had been continuously publishing the study results of optical method for tip clearance measurement [8,9,10,11,12]. Meanwhile, the study of Andreas K. et.al. [13] proved the optical sensor still had some problems to be solved such as optical fiber heat-resistance, lens cleanness, and the Doppler effect. Capacitive method is the most mature technology so far. Early as 1953, Mossop I.A. et al. [14] published a set of capacitive system for turbine tip clearance measurement. Muller D. et al. [15] conducted the dynamic tip clearance measurement experiment on the compressor and turbine and validated the system uncertainty and stabilization, while the sensor may be influenced by permittivity change of medium and has zero drift problems. Chana K.S. and Sridhar V. et al. [16,17,18,19] used an eddy current probe on the gas turbine engine to obtain tip clearance values of the high-pressure turbine stage. The results showed the sensor was able to perform at these extreme environments without losing accuracy. Du L. and Zhu X.L. et al. [20,21] verified the eddy current method feasibility in laboratory with 3000 rpm speed at 1300 K temperature.

Based on the existing studies, inductive sensors have been developed and have gained considerable success for their simple structure, low cost, and easy installation [20]. It is immune to contaminations when compared with industry standard optical probes [17] and offers the possibility of being able to take blade passing data through the casing [22,23]. The inductive method is potential to monitor the dynamic blade tip clearance and blade arrival timing simultaneously.

However, the measurement signal of eddy current sensor has temperature drift problems and it is inevitably affected by temperature variation for the reason of measuring principle. Lyu Y.T. et al. [24] used temperature-compensation circuit to eliminate temperature drift of inductive sensor from 20 °C to 500 °C. The experimental research of Wang H.B. et al. [25] indicated the resistance has a larger temperature coefficient than the inductance. Zhao Z.Z. et al. [26,27] focused on the eddy current sensor design and validation. The simulation and experiment method were both used to determine the optimal sensor structure parameters and materials. The designed sensor was tested at extremely high temperature to prove it can withstand the actual operating temperature. In the recent study [28], the relation between temperature and sensor parameters has been explored. When the temperature varied from 50 °C to 1100 °C, the sensor resistance changed with temperature significantly while inductance was almost unchanged.

The current research mainly focuses on the temperature compensation and practical application, so there is little experimental research of temperature effect on the sensor performance especially on the characteristic curve. As the basis of measurement, accurate calibration is beneficial to optimize the measurement uncertainty. Hence, this paper focused on validating the high-resolution inductive sensor performance and aimed at finding the temperature influence on the sensor characteristics and measurement results in the range of 600 °C to 1000 °C. Furthermore, instead of the signal processing circuits in [28], the simpler voltage division circuit was used to measure the clearance variation signal. The sensor repeatability error was verified by repeated calibration and then the measurement error at different temperatures was obtained by calibration at different temperature. Finally, the temperature influence on sensor performance was proved could be ignored by comparing the repeatability error and measurement error at different temperatures.

## 2. Method and Sensor

### 2.1. Inductive Tip Clearance Measurement

**Figure 1:**
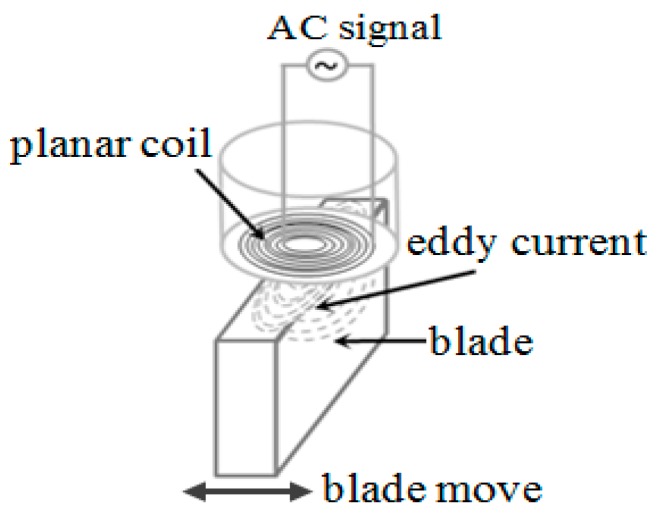
Schematic diagram of measuring principle.

The working principle of inductive sensor is based on Faraday’s law of electromagnetic induction and Lenz’s law. The main component of the sensor is an inductive coil, which can be a three-dimensional spiral coil or a two-dimensional planar coil. The coil generates a magnetic field when excited by a high frequency AC signal, and then the eddy current is induced in the metallic target when it passes through the magnetic field and thus a reverse magnetic flux caused by eddy current declines the inductance of sensor coil. Figure 1 shows the working principle of the inductive sensor (encapsulated planar coil).

**Figure 2:**
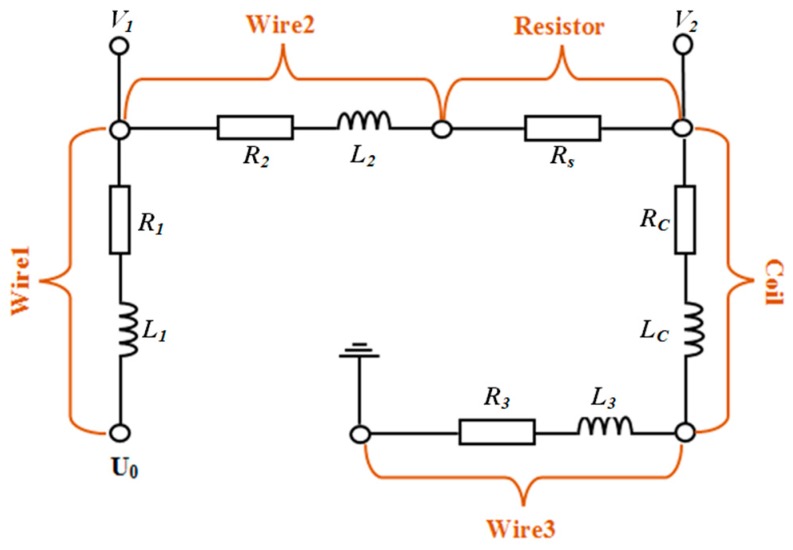
Equivalent circuit of measurement system.

Figure 2 is the measurement system equivalent circuit. Wire1 is the signal wire. Wire2 and Wire3 are the connecting wires. Resistor is the voltage divider in the measurement circuit. The connecting wire in high frequency circuit can be equivalent to series resistance and inductance in general. And sensor coil can also be equivalent to series resistance and inductance.

The relation between equivalent circuit parameters and output signal *V_1_* and *V_2_* can be derived from the Kirchhoff Voltage Law (Equations (1) and (2)).

(1) $$I*\left[\left(R_{2}+R_{s}+R_{c}+R_{3}\right)+j\omega \cdot \left(L_{2}+L_{c}+L_{3}\right)\right]=V_{1}$$

(2) $$I*\left[\left(R_{c}+R_{3}\right)+j\omega \cdot \left(L_{c}+L_{3}\right)\right]=V_{2}$$

wherein, *I* is the current in the circuit, $R_{s}$ is the divider resistance, $R_{c}$ and $L_{c}$ are equivalent resistance and inductance of coil. $R_{2}$ and $R_{3}$ are the equivalent resistance of circuit, and Wire2 and Wire3 are connection wires of sensor, series resistance, and data acquisition card. $L_{2}$ and $L_{3}$ are the equivalent inductance of Wire2 and Wire3. $R_{2}$, $R_{3}$, $L_{2}$, $L_{3}$, and $R_{s}$ should be measured in advance before measurement and substituted as known quantities into data processing program to calculate the divided voltage on the sensor coil *Vc*. Inductance of connecting wires in the circuit were much smaller than $L_{c}$, so $L_{2}$ and $L_{3}$ can be ignored, and $R_{2},R_{3}$ were measured by LCR (inductance, capacitance and resistance) meter both as 0.86 Ω, while $R_{s}$ was measured as 10.1 Ω.

The current in the circuit I can be derived from Equations (1) and (2) as Equation (3) shows and the divided voltage on the sensor coil (*V_C_*) is Equation (4):

(3) $$I=\frac{V_{1}-V_{2}}{R_{2}+R_{s}}$$

(4) $$V_{C}=V_{2}-I*R_{3}$$

**Figure 3:**
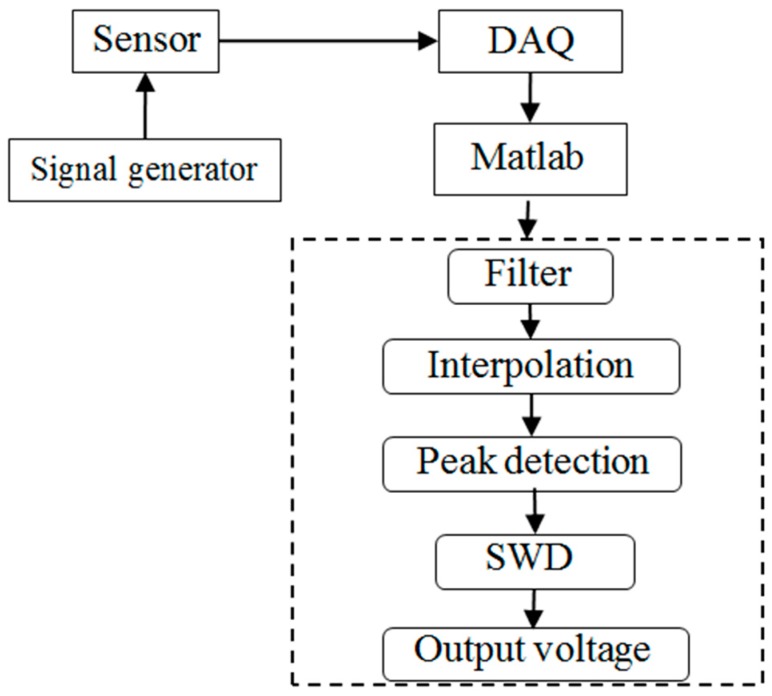
Measurement data processing process. SWD: stationary wavelet decomposition. DAQ: data acquisition.

Data processing process is shown in Figure 3. The signal was collected by DAQ (Data Acquisition) and then input into Matlab platform. In order to increase the signal-to-noise ratio (SNR), FFT method was used to remove the high frequency noise and stationary wavelet decomposition (SWD) method was used to smooth the voltage signal.

**Figure 4:**
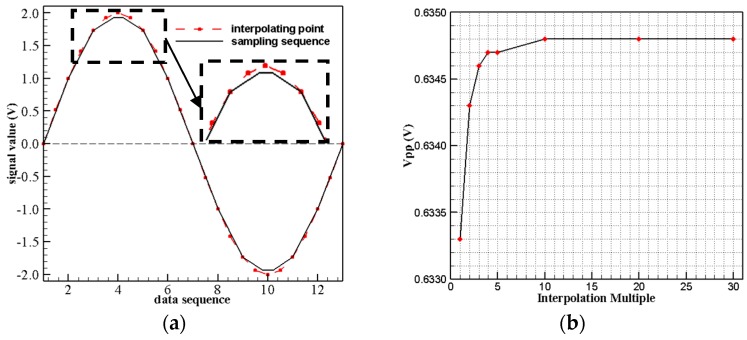
(**a**) Peak error caused by signal discontinuity; (**b**) Data interpretation effect comparison.

There is peak detection error caused by the discontinuity of collection signal as Figure 4a shows. So, the interpolation is used to increase the peak detection accuracy. Figure 4b is the comparison of the peak value with different interpolation multiples. It is proved that interpolation can efficiently increase the accuracy of peak detection and the tenfold interpolation is sufficient to obtain the effective peak result.

### 2.2. Sensor Structure and Manufacture

The magnetic field intensity of circular coil is higher than that of square coil. The magnetic field intensity *B* generated by circular coil is calculated by Equation (5). When the material permeability μ is determined by material property and current *I* is determined by exciting circuit, it can be seen that coil turns (*N*) and coil thickness (*h*) can both influence the magnetic field intensity.

(5) $$B=\mu \frac{N\times I}{h}$$

Harold Wheeler [29] had published the research results of eddy current coil in 1928 and Equation (6) is the planar coil inductance formula. It indicates the coil inductance also has positive correlation with coil turns (*N*), radial dimension (*r*), and inverse correlation with thickness (*h*).

(6) $$L\left(\mu H\right)=\frac{r^{2}\times N^{2}}{\left(8r+279.4h\right)}$$

From the above, the magnetic field intensity and coil inductance are proportional to *N* and inversely proportional to *h* when the sensor coil is connected to a certain circuit. That is to say the coil with more turns and thinner thickness can generate higher magnetic field intensity, thus coil has higher sensitivity and wider measuring range.

The study result of Du L. et al. [22] indicated the planar coil had simple geometry, intensive magnetic field, and fast response speed, and its sensitivity and range satisfied the turbine tip clearance measurement requirement. Thus, the sensor in this paper is also planar coil without iron core, that is to say the minimum *h* equals to the wire diameter and the compact method is adopted to wrap coils to permit more turns for the given coil size and generate higher magnetic flux density.

**Figure 5:**
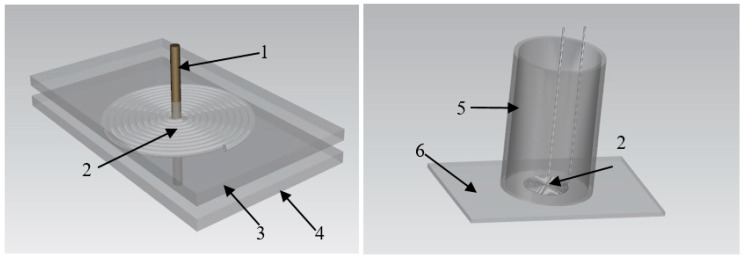
Sensor making device, wherein **1** is central tube, **2** is planar coil, **3** is upper plate, **4** is bottom plate, **5** is ceramic gelcontainer, **6** is fixed plate.

The sensor probe in this paper was manufactured by the device as Figure 5 shows. According to the study results in [27,28], the planar coil was made of 0.3 mm platinum wire which melting point is over 2000 K. The platinum wire was in advance painted by insulating paint which is heat-resistant of 1050 °C to prevent the shortage between windings. First, drilled 1.2 mm diameter holes in the center of two separate 3 cm × 4 cm acrylic plates and then fixed them in parallel and kept the distance slightly over 0.3 mm. Then, a 0.8 mm diameter tube was inserted through the holes and fixed well. The wire was wrapped around the tube between the two plates to form the planar hollow coil. When the planar coil was formed, carefully removed the central tube and the upper plate, and then thin coil was glued off the bottom plate by adhesive tape. Finally, a high temperature ceramic adhesive gel was used to seal the coil to avoid it being corroded at corrosive environment.

**Figure 6:**
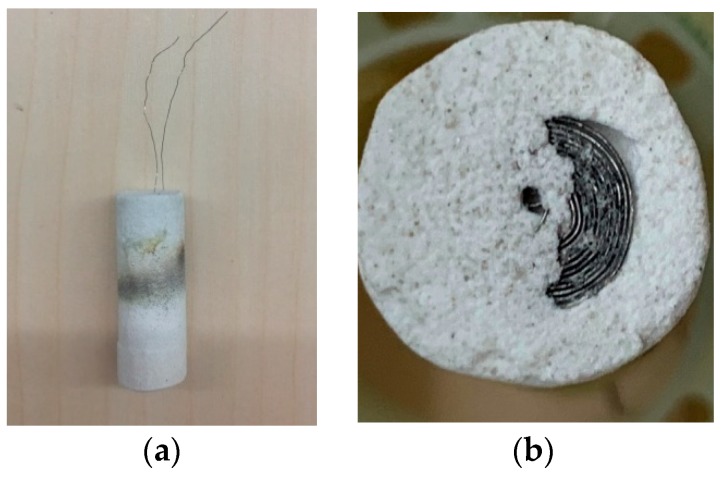
(**a**) Encapsulated sensor; (**b**) Sensor Coil in the encapsulation.

Three coils were fabricated by the method above with the same structure and the parameters were measured by the LCR meter at 4 MHz. Table 1 listed the measurement results. *Q* is the quality factor which indicates the inductive element quality and it is equals to $\frac{\omega L_{c}}{R_{c}}$. Their parameters indicated the coils shared the similar property so the first coil was used in the paper which was an encapsulated 10-turns coil as Figure 6 shows.

For the phase angle that indicates the inductive element quality, which means the higher phase angle, the element is more inductive and more sensitive in the remote inductive measurement. Equation (7) is the calculation formula of the sensor phase angle.

(7) $$phase\;angle=\arctan\left(\frac{\omega L_{c}}{R_{c}}\right)$$

wherein, ω is the circuit angular frequency, $L_{c}$ is the coil inductance, and $R_{c}$ is the coil resistance.

**Figure 7:**
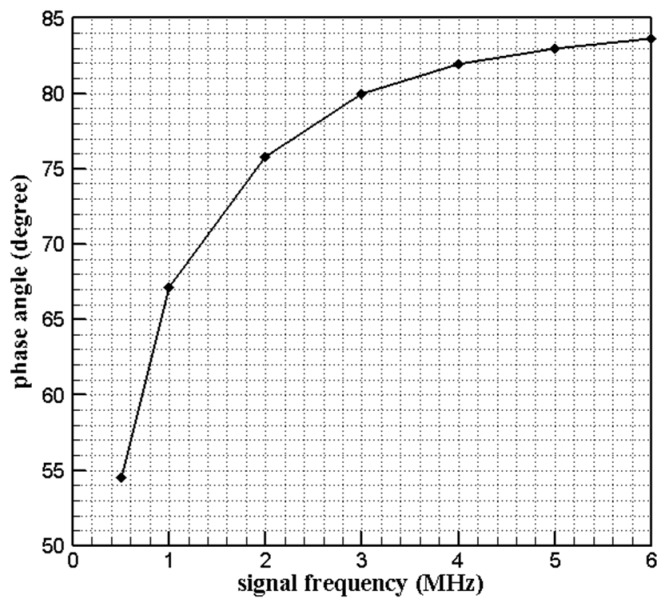
Sensor phase angle varies with excitation frequency.

In order to determine the signal frequency in the experiment and the quality of the sensor, the phase angle of sensor at different frequency were measured by LCR meter and Figure 7 shows the result. The sensor phase angle is over 80° when the frequency is over 4 MHz from Figure 7 so the frequency was determined as 4 MHz.

## 3. Sensor Performance Calibration

### 3.1. Characteristics Calibration

In order to verify the sensitivity and measuring range of manufactured sensor, the calibration experiment was conducted at room temperature to obtain the sensor characteristic curve. The characteristic curve of the tip clearance sensor refers to the relationship curve of sensor characteristic parameter and the clearance. The relative variation of voltage was used as measuring quantity in the study.

**Figure 8:**
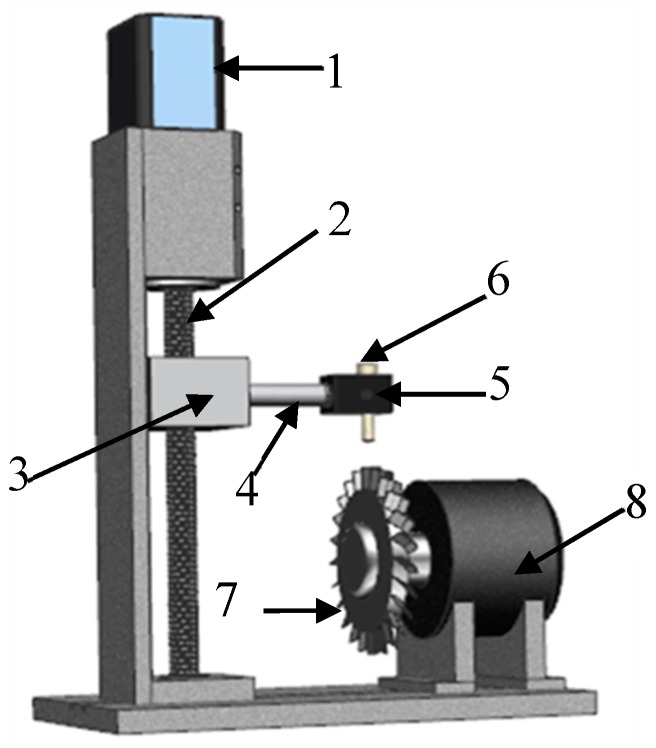
Calibration setup. 1-stepping motor, 2-screw, 3-slider, 4-spindle extension, 5-clip, 6-sensor, 7-target, 8-driving motor.

The calibration device, as Figure 8 shows, mainly includes position controller and rotator. The displacement slider is driven by the screw which is controlled by stepping motor. The precision of position controller is 1 μm and its control range is 13 mm. The calibration target is an 18 mm width and 1.5 mm thickness blade, and the target material is Inconel 718 which is one of the turbine blade materials. The excitation signal in the calibration experiment was 4 MHz and 3 Vpp sinusoidal AC signal generated by Agilent Keysight 33600A (Agilent Technologies Inc., Santa Clara, CA, USA).

The calibration system includes sensor probe, calibration target, position controller, function generator, and DAQ system. After connecting the measuring circuit (function generator, sensor probe and DAQ card) and fixing the target position, we collected the voltage V_0_ on the sensor coil without target object. Then, we adjusted the distance between sensor surface and target blade surface from 0 mm to 5 mm with 50 μm step, and collected voltage signals on the sensor coil at each position. The voltage relative variation dV/V_0_ was denoted as sensor characteristic parameter and calculated following the steps shown in Figure 3.

**Figure 9:**
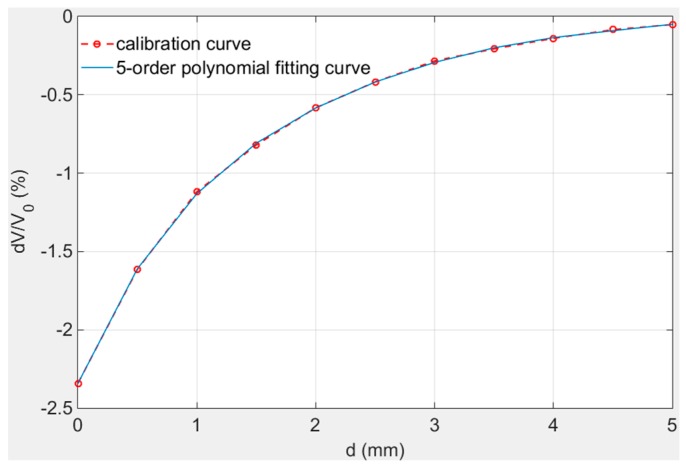
Calibration curve at room temperature.

The calibration result is shown in Figure 9. The measurement curve was composed of 11 measuring points, therefore, a suitable formula is needed to express the curve in the full range. The five-order fitting curve has the best fitting degree and it represented the calibration result smoothly. Other lower order fitting curve may produce errors in individual positions. Equation (8) is five order fitting formula of calibration curve and its fitting degree R^2^ is 0.9992. The measurement data near 5 mm is listed in Table 2.

(8) $${\mathrm{dV}/V}_{0}\;=\;a_{0}\;+\;a_{1}\;\times \;d\;+\;a_{2}\;\times \;d^{2}+\;a_{3}\;\times \;d^{3}+\;a_{4}\;\times \;d^{4}+a_{5}\;\times \;d^{5}$$

wherein, a_0_ = −2.3419; a_1_ = 1.7963; a_2_ = −0.76074; a_3_ = 0.20458; a_4_ = −0.030464; a_5_ = 0.0018549. According to the calibration curve in Figure 9, the sensor has good sensitivity (1%/mm) within 2 mm. The sensitivity and the resolution of the sensor decrease with the distance as Figure 9 shows and the data resolution of measurement system is 0.0001%. Data listed in Table 2 proves the measuring range of the sensor is over 5 mm and the resolution reaches 10 μm for the data still has 0.001% variation at 5 mm position.

**Figure 10:**
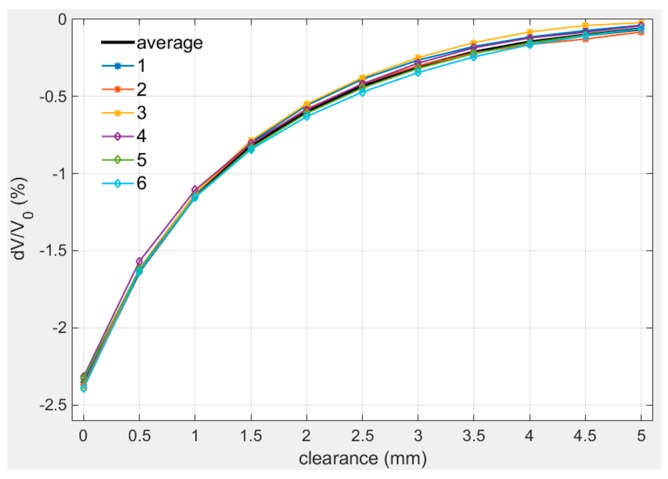
Repeated calibration curves at room temperature.

In order to verify the repeatability of the sensor, three sets of repeated calibration experiments were carried out at room temperature: collected the measured signals of the sensor at different positions with 50 μm step length from 0 mm to 5 mm, and then collected the signals from 5 mm to 0 mm in the same way. Repeated this process three times and recorded the results as Figure 10 shows. The average value of six measured value was plotted as average curve. The range of three sets of calibration values varied from −2.5% to 0% and the variation within 0 mm–2 mm was closed to 1%/mm.

The fitting parameters of different calibration curves are listed in Table 3. The data in Table 3 proves the calibration curves share the same characteristics.

**Figure 11:**
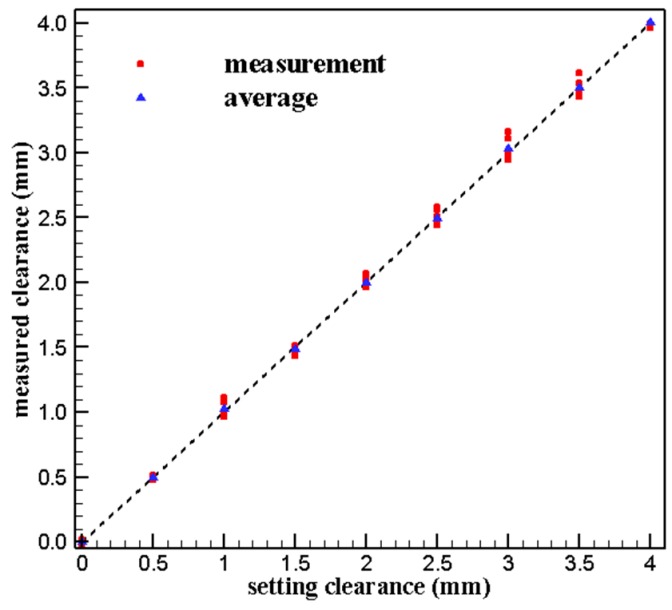
Results of repeated measured.

The clearance measurement results at different positions are plotted in Figure 11 and the relative error of measurement results are listed in Table 4. It can be seen that the measurement error is smaller within 2.5 mm while it is larger over 3 mm, especially at 3 mm, the relative error reaches the maximum of 5.4%.

### 3.2. Calibration at High Temperature

**Figure 12:**
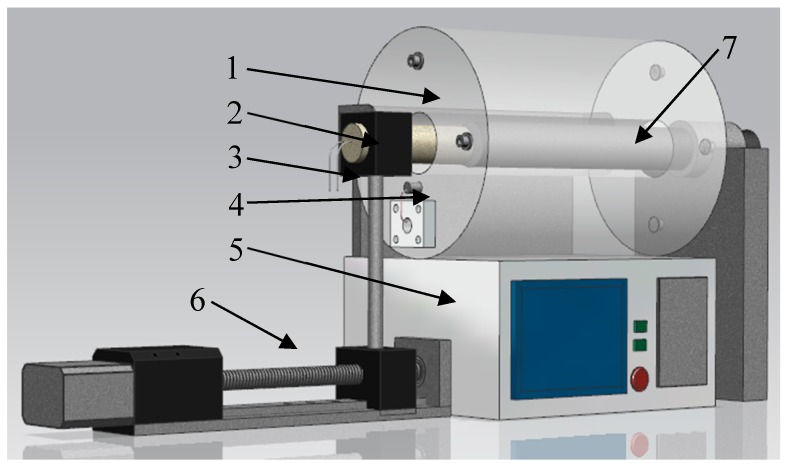
Hightemperature calibration setup. (1—heater,2—sensor,3—sensor lead wire, 4—thermocouple, 5—temperature controller, 6—position adjuster, 7— target).

The purpose of calibration at high temperature is validating the sensor so it can operate well at different environment temperatures over 500 °C. The heat resistance of the sensor was verified in [28]. The high temperature calibration was conducted with the device as Figure 12 shows. The device was consisted with a tubular heater, a high precision position adjuster, the sensor, and the target object.

The heater temperature control precision is 1 °C by PID control method and the position adjuster with ±1 μm accuracy is controlled through the motor driving screw.

**Figure 13:**
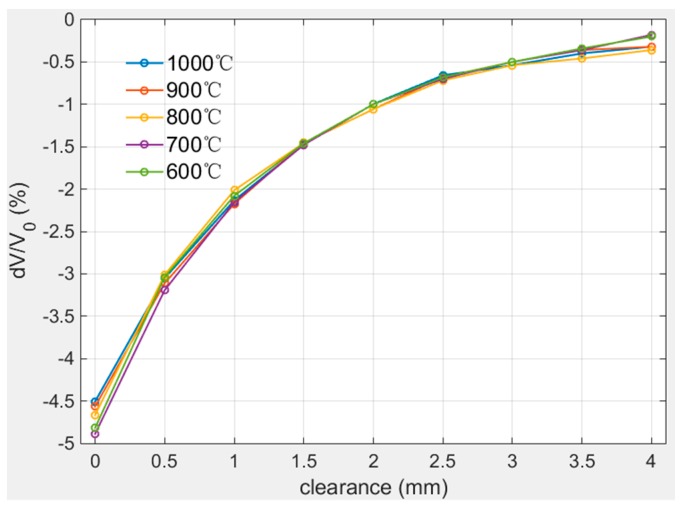
Calibration curves at high temperatures.

The calibration experiments at high temperatures were carried out in the range of 600 °C–1000 °C and the calibration range was 0 mm–4 mm with 50 μm step length. The calibration results are plotted in Figure 13 and as it shows, excepting the start point 0 mm and end point 4 mm, the curves have high coincidence especially in the range of 1 mm–3 mm. The range of calibration values at different temperature varied from −5.0% to 0% and the variation within 0 mm–2 mm was closed to 2.0%/mm.

The fitting parameters of different calibration curves are listed in Table 5. The data in Table 5 proves the calibration curves share the same characteristics.

**Figure 14:**
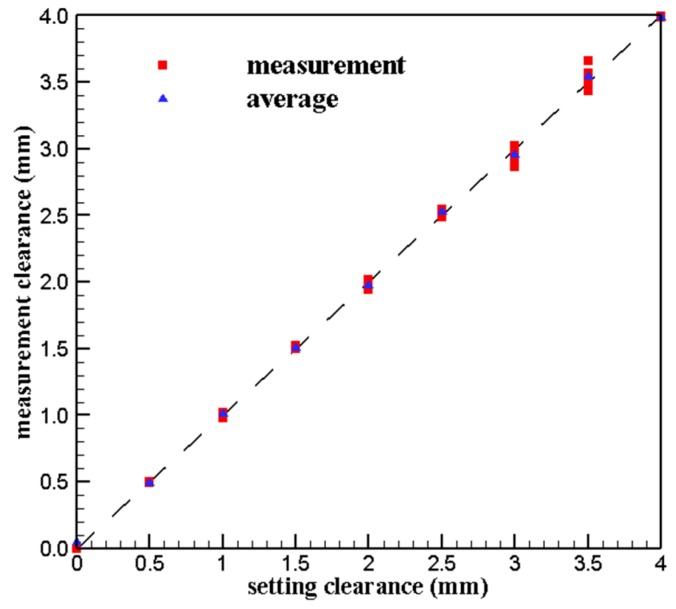
Measurement accuracy error at high temperatures.

The clearance measurement results of different temperature at different positions are plotted in Figure 14 and the relative error of measurement results are listed in Table 6. It can be seen that the measurement error is smaller within 2.5 mm while it is larger over 3.0 mm, especially at 3.0 mm, the relative error reaches the maximum of 4.6%.

Compared with the repeated calibration results at room temperature in Section 3.1, the calibration results at high temperatures have smaller relative errors, so it can be considered that the sensor calibration results at different high temperatures are consistent.

### 3.3. Results Discussion

According to the calibration results at room temperature, the measuring range and resolution of designed sensor were proved to be over 5 mm and 10 μm respectively. The maximum relative error of repeated measurement was 5.4% within the range of 4 mm based on the analysis of results of repeated calibration experiments.

The error analysis of calibration results at different high temperatures shows that the maximum relative error of the 600 °C–1000 °C calibration results was 4.6%, which is smaller than that of the repeated measurement. This indicates the difference of measurement results during the range of 600 °C–1000 °C may be caused by the sensor repeatability error, and the temperature effect on the measurement results can be ignored at high temperature. This conclusion is consistent with the conclusion in [28] that the influence of temperature on sensor inductance can be ignored for the variable of distance measuring of inductive sensor is inductance.

The above conclusions apply to the planar platinum wire coil which is fabricated with the same method and has the similar structure with the coil in this paper. While the material or the structure is changed, the coil inductance may change and also the resistance of other materials may change differently with temperature, so the coil performance at the high temperature should be tested additionally.

## 4. Conclusions

Based on the principle of inductive clearance measurement sensor and comparative experimental research, the following conclusions can be drawn in this paper:

1. The designed sensor with planar coil made of platinum wire was proved to be a good inductive sensor for its phase angle is up to 80° under 4 MHz excitation frequency and the sensor performance meets the requirements of tip clearance measurement for the measuring range was over 5 mm and the resolution is better than 0.01 mm within 5 mm range according to the static calibration result.
2. The sensor was effective at high temperature like 1000 °C, and maintains good stability within the temperature range from 600 °C to 1000 °C. This suggests the designed sensor is capable to operate over a wide temperature range and that is an important basis for the sensor to be used in turbine tip clearance measurement in the future.
3. Compared with the measurement relative error of repeated calibration at room temperature, that of measurement under different temperatures was smaller, which means the difference of measurement value at different temperature may be caused by repeatability error rather than temperature influence. This meaningful conclusion will greatly simplify the calibration process and data process at high temperature.

The measurement system is usually cooled by additional cooling system. The temperature range investigated in this paper is the temperature that current sensor can withstand not the real turbine temperature.

Furthermore, it is necessary to conduct in-depth research work on reducing the sensor repeatability error in the future. After that, a series of thermal dynamic tests will be conducted on the turbine rotor using the measurement method adopted in this paper.

## Figures and Tables

**Table 1:** Parameters of three sample coils.

No.	*L* (μH)	*R* (Ω)	*Q*	*θ* (°)
1	0.366	1.22	7.7	82.6
2	0.359	1.25	7.2	82.1
3	0.361	1.27	7.1	82.0

**Table 2:** Calibration data at near full range area.

Clearance(mm)	dV/V_0_ (%)
4.95	−0.0543
4.96	−0.0534
4.97	−0.0525
4.98	−0.0515
4.99	−0.0506
5.00	−0.0496

**Table 3:** Repeated calibration fitting curve parameters.

Stroke	a_0_	a_1_	a_2_	a_3_	a_4_	a_5_
1	−2.3495	1.7250	−0.6444	0.1508	−0.0203	0.0012
2	−2.3745	1.8665	−0.8139	0.2254	−0.0349	0.0022
3	−2.3921	1.8433	−0.7353	0.1799	−0.0239	0.0013
4	−2.3162	1.8907	−0.9259	0.2906	−0.0484	0.0032
5	−2.3288	1.7326	−0.7154	0.1870	−0.0269	0.0016
6	−2.3908	1.8871	−0.8533	0.2357	−0.0344	0.0020
average	−2.3721	1.8252	−0.7839	0.2134	−0.0320	0.0020

**Table 4:** Measurement error.

d (mm)	Error (%)
0.5	4.00
1.0	3.10
1.5	4.23
2.0	3.50
2.5	3.32
3.0	5.40
3.5	3.34
4.0	3.90

**Table 5:** Calibration fitting curve parameters at different temperature.

Temperature (°C)	a_0_	a_1_	a_2_	a_3_	a_4_	a_5_
600	−4.8154	4.7364	−2.9403	1.1855	−0.2555	0.0217
700	−4.8883	4.3053	−2.1853	0.7583	−0.1551	0.0133
800	−4.6701	4.4476	−2.6413	1.0159	−0.2111	0.0174
900	−4.5647	3.5156	−1.4324	0.3890	−0.0623	0.0043
1000	−4.5040	3.6239	−1.7676	0.6586	−0.1485	0.0135

**Table 6:** Measurement error at high temperatures.

d (mm)	Error (%)
0.5	1.40
1.0	1.90
1.5	1.80
2.0	2.80
2.5	1.96
3.0	4.60
3.5	4.49
4.0	0.35
